# Shiga Toxin-Mediated Hemolytic Uremic Syndrome: Time to Change the Diagnostic Paradigm?

**DOI:** 10.1371/journal.pone.0001024

**Published:** 2007-10-10

**Authors:** Martina Bielaszewska, Robin Köck, Alexander W. Friedrich, Christof von Eiff, Lothar B. Zimmerhackl, Helge Karch, Alexander Mellmann

**Affiliations:** 1 Institute of Hygiene and the National Consulting Laboratory on Hemolytic Uremic Syndrome, University of Münster, Münster, Germany; 2 Institute of Medical Microbiology, University of Münster, Münster, Germany; 3 Department of Pediatrics, University Hospital of Innsbruck, Innsbruck, Austria; University of California at Merced, United States of America

## Abstract

**Background:**

Hemolytic uremic syndrome (HUS) is caused by enterohemorrhagic *Escherichia coli* (EHEC) which possess genes encoding Shiga toxin (*stx*), the major virulence factor, and adhesin intimin (*eae*). However, the frequency of *stx*-negative/*eae*-positive *E. coli* in stools of HUS patients and the clinical significance of such strains are unknown.

**Methodology/Principal Findings:**

Between 1996 and 2006, we sought *stx*-negative/*eae*-positive *E. coli* in stools of HUS patients using colony blot hybridization with the *eae* probe and compared the isolates to EHEC causing HUS. *stx*-negative/*eae*-positive *E. coli* were isolated as the only pathogens from stools of 43 (5.5%) of 787 HUS patients; additional 440 (55.9%) patients excreted EHEC. The majority (90.7%) of the *stx*-negative/*eae*-positive isolates belonged to serotypes O26:H11/NM (nonmotile), O103:H2/NM, O145:H28/NM, and O157:H7/NM, which were also the most frequent serotypes identified among EHEC. The *stx*-negative isolates shared non-*stx* virulence and fitness genes with EHEC of the corresponding serotypes and clustered with them into the same clonal complexes in multilocus sequence typing, demonstrating their close relatedness to EHEC.

**Conclusions/Significance:**

At the time of microbiological analysis, ∼5% of HUS patients shed no longer the causative EHEC, but do excrete *stx*-negative derivatives of EHEC that lost *stx* during infection. In such patients, the EHEC etiology of HUS is missed using current methods detecting solely *stx* or Shiga toxin; this can hamper epidemiological investigations and lead to inappropriate clinical management. While maintaining the paradigm that HUS is triggered by Shiga toxin, our data demonstrate the necessity of considering genetic changes of the pathogen during infection to adapt appropriately diagnostic strategies.

## Introduction

Hemolytic uremic syndrome (HUS) consists of microangiopathic hemolytic anemia, thrombocytopenia, and renal insufficiency [1]. It usually develops after prodromal diarrhea, which is often bloody [1], [2]. HUS is a leading cause of acute renal failure in children [3] and the mortality during the acute phase reported in recent studies was ∼2% [4]–[6]; many survivors suffer from renal or non-renal sequelae [1], [3].

The major etiological agents of HUS are enterohemorrhagic *Escherichia coli* (EHEC) strains belonging to serotype O157:H7 and several other serotypes, including O26:H11/NM (nonmotile), O103:H2/NM, O111:H8/NM, O145:H28/NM, and O157:NM [1], [2], [4]–[11]. The cardinal virulence traits of EHEC are Shiga toxins (Stx) [12], which cause microvascular endothelial injury in kidneys and other organs resulting in the characteristic thrombotic microangiopathy that forms the histopathological basis of HUS [1], [13]. Stx production is mediated by lysogenic conversion of EHEC with *stx*-harboring prophages, which integrate into specific sites in their chromosomes [14]–[16]. These phages can be excised by treatment with UV light, antibiotics, or by various stimuli in the host [14], [16]–[18]. The majority of EHEC strains associated with HUS also harbor the *eae* gene encoding intimin [8], [19], which mediates intimate attachment of the bacteria to the intestinal mucosa [20].

Although HUS is typically caused by EHEC, *stx*-negative/*eae*-positive (*stx*−/*eae*+) *E. coli* strains are occasionally excreted by patients with HUS [21], [22]. However, the frequency of such strains is unknown, and their origins and clinical significance are poorly understood. To answer these questions, we studied stools from HUS patients, processed so as to detect these variants. We characterized the identified isolates and compared them to EHEC associated with HUS with respect to serotypes, virulence and fitness genes, phenotypes, and multilocus sequence types.

## Results

### Frequency and serotypes of *stx*−/*eae*+ *E. coli* in stools from patients with HUS

Between 1996 and 2006, *stx*−/*eae*+ *E. coli* strains were isolated from stools of 43 (5.5%) of 787 individual, epidemiologically unrelated HUS patients; additional 440 (55.9%) patients excreted EHEC (Table 1), resulting in an overall isolation rate of 61.4% (483 of 787). In none of the 483 culture-positive patients *stx*-negative and EHEC strains were found together in the same stool. Thirty-nine (90.7%) of the *stx*−/*eae*+ isolates belonged to serotypes O26:H11/NM, O103:H2/NM, O145:H28/NM, and O157:H7/NM (Table 1), which also accounted for the majority (91.1%) of the EHEC isolates (Table 1). One additional *stx*−/*eae*+ strain belonged to serotype O121:H19 (which was also found among EHEC; Table 1), and the remaining three were nontypeable (Table 1). Each of the 43 *stx*−/*eae*+ strains lacked all known *stx* alleles. The stools from which these strains were isolated contained neither *stx* genes as demonstrated by PCR screening of enriched primary stool cultures, nor free Stx as demonstrated by the Vero cell assay on stool filtrates, nor any classic bacterial enteric pathogens including *Salmonella*, *Shigella*, and *Yersinia* spp., and *Campylobacter jejuni*.

**Table 1:** Numbers of HUS patients from whom *stx*-negative/*eae*-positive *E. coli* or EHEC strains were isolated and serotypes of the isolates.

Serotype^a^	Patients with *stx*−/*eae*+ isolates		Patients with EHEC isolates	
	Number of patients	Percentage Total	Number of patients	Percentage Total
O26:H11/NM	13	30.2	58	13.2
O103:H2/NM	4	9.3	15	3.4
O111:H8/H10/NM	0	0	11	2.5
O145:H28/NM	5	11.6	31	7.0
O157:H7/NM (NSF)	2	4.7	221	50.2
O157:NM (SF)	15	34.9	76	17.3
Others	4^b^	9.3	28^c^	6.4
Total	43^d^	100	440^d^^,^ e	100

a NM, nonmotile; NSF, non-sorbitol-fermenting; SF, sorbitol-fermenting.

b Serotypes (number of isolates, if more than one, in parenthesis): O121:H19, ONT:H6 (2), ONT:H7; ONT, O antigen not typeable with antisera against *E. coli* O antigens 1 to 181.

c Serotypes (number of isolates, if more than one, in parenthesis): O4:NM, O55:H7, O55:HNT, O70:H8, O73:H18, O76:H19, O91:H21 (2), O98:NM, O104:H4, O112:NM, O113:H21 (2), O119:H2, O121:H19 (2), O128:H2, O136:HNT, O145:H25 (2), O163:H19, O174:H21, Orough:H2, Orough:H11, Orough:NM, ONT:H21, ONT:NM, ONT:HNT; Orough, autoagglutinable strains; HNT, H antigen not typeable with antisera against *E. coli* H antigens 1 to 56.

d In none of the 483 culture-positive patients *stx*-negative and s*tx*-positive (EHEC) strains were found in the same stool sample.

e Four patients shed two different EHEC serotypes including O157:H7 and O145:NM; O157:H7 and O103:H2; SF O157:NM and O145:NM; O26:H11 and O145:NM (the underlined serotypes which prevailed in the stools and were isolated as the first are included in the table).

### Molecular characteristics of *stx*−/*eae*+ *E. coli* isolates

We compared the *stx*−/*eae*+ *E. coli* O26:H11/NM, O103:H2/NM, O121:H19, O145:H28/NM and O157:H7/NM isolated from HUS patients to randomly selected HUS-associated EHEC isolates of corresponding serotypes for the presence of several genes that are known to be typically distributed in EHEC [9], [11], [23]–[29] (Table 2). The *stx*−/*eae*+ strains of each serotype closely resembled EHEC of the corresponding serotype with respect to the presence or absence of putative non-*stx* virulence genes encoding toxins (EHEC-*hlyA*, *cdt-V*), adhesins (*iha*, *lpfA*_O26_, *lpfA*_O157/OI 141_, *lpfA*_O157/OI 154_, *sfpA*), and virulence determinants of the O island 122 of *E. coli* O157:H7 (*efa1*, *sen*, *pagC*), as well as the *ter* gene cluster and the *irp2* and *fyuA* components of an iron uptake system (Table 2). Moreover, the *stx*-negative and *stx*-positive strains within each serotype shared the *eae* type and *fliC* gene encoding the flagellin subunit of the H antigen (Table 2). Consistently with the absence of *stx*, the chromosomal loci which serve as integration sites for *stx*-converting bacteriophages in EHEC O157:H7/NM (*yehV*, *wrbA*, *yecE*) [14], [15] and EHEC O26:H11/NM (*wrbA*, *yecE*) [16] were unoccupied in each of the *stx*−/*eae*+ strains of the respective serotypes. This suggests that the absence of *stx* in these strains was associated with the excision of *stx*-harboring phages from their chromosomes.

**Table 2:** Comparison of putative virulence and fitness genes of *stx*-negative and *stx*-positive *E. coli* strains of serotypes O26:H11/NM, O103:H2/NM, O121:H19, O145:H28/NM, and O157:H7/NM.

Gene or gene cluster^a^	Predicted product or phenotype^b^	Presence of the gene among *stx*-negative and *stx*-positive *E. coli* strains of serotype^c^											
		O26:H11/NM		O103:H2/NM		O121:H19		O145:H28/NM		O157:H7/NM (NSF)		O157:NM (SF)	
		*stx*−	*stx*+	*stx*−	*stx*+	*stx*−	*stx*+	*stx*−	*stx*+	*stx*−	*stx*+	*stx*−	*stx*+
		(n = 13)	(n = 15)	(n = 4)	(n = 8)	(n = 1)	(n = 2)	(n = 5)	(n = 10)	(n = 2)	(n = 10)	(n = 15)	(n = 20)
EHEC-*hlyA*	EHEC hemolysin	+	+	+	+	+	+	+	+	+	+	+	+
*cdt-V*	CDT-V	−	−	−	−	−	−	−	−	−	−	+ (87)	+ (85)
*iha*	Iha	+ (92)	+ (93)	−	−	−	−	+	+	+	+	−	−
*lpfA*_O26_	LpfA_O26_	+	+	+	+	−	−	−	−	−	−	−	−
*lpfA*_O157/OI 141_	LpfA_O157_ (OI 141)	−	−	−	−	−	−	+	+	+	+	+	+
*lpfA*_O157/OI 154_	LpfA_O157_ (OI 154)	−	−	−	−	−	−	−	−	+	+	+	+
*sfpA*	Sfp fimbriae	−	−	−	−	−	−	−	−	−	−	+	+
*efa1*	Efa1	+^d^	+^d^	+^d^	+^d^	+^d^	+^d^	+^d^^,^ e	+^d^^,^ e	+^e^	+^e^	+^d^	+^d^
*sen*	ShET2 homologue	+	+	+	+	+	+	+ (80)	+ (90)	+	+	+	+
*pagC*	PagC homologue	−	−	−	−	+	+	+ (20)	+ (10)	+	+	+	+
*ter* f	Tellurite resistance	+ (92)	+ (93)	+ (25)	+ (13)	+	+	+	+	+	+	−	−
*irp2/fyuA* g	Iron uptake	+	+	−	−	−	−	−	−	−	−	−	−
*eae* (type)	Intimin	+ (β)	+ (β)	+ (ε)	+ (ε)	+ (ε)	+ (ε)	+ (γ)	+ (γ)	+ (γ)	+ (γ)	+ (γ)	+ (γ)
*fliC* h	Flagellin subunit	H11	H11	H2	H2	n.p.	n.p.	H28	H28	H7	H7	H7	H7

a The genes were detected by PCR as described in Materials and Methods. GenBank accession numbers: EHEC-*hlyA* (NC007414); *cdt-V* (AJ508930); *iha* (AF126104; AE005174); *lpfA*_O26_ (AB161111), *lpfA*_O157/OI 141_ (AE005174), *lpfA*_O157/OI 154_ (AE005174), *sfpA* (NC009602), *efa1* (AE005174; AJ459584), *sen* (AE005174), *pagC* (AE005174), *ter* cluster (AE005174), *irp2*, *fyuA* (NC003143), *eae* (AE005174).

b EHEC, enterohemorrhagic *E. coli*; CDT-V, cytolethal distending toxin V; Iha, iron-regulated gene A homologue adhesin; LpfA_O26_ and LpfA_O157_, major fimbrial subunits of long polar fimbriae of EHEC O26 and EHEC O157, respectively; LpfA_O157_ (OI 141) and LpfA_O157_ (OI 154), LpfA encoded on O island (OI) 141 and OI 154, respectively, of *E. coli* O157:H7 strain EDL933; Sfp, sorbitol-fermenting EHEC O157, plasmid-encoded; Efa1, EHEC factor for adherence; ShET2, *Shigella flexneri* enterotoxin 2; PagC, protein encoded by the *phoP*-activated gene C (*pagC*) of *Salmonella enterica* serovar Typhimurium.

c NM, nonmotile; NSF, non-sorbitol-fermenting; SF, sorbitol-fermenting; *stx*−, *stx*-negative; *stx*+, *stx*-positive; n, number of strains tested; +, all strains tested (n) were positive for the gene; −, all strains tested (n) were negative for the gene; if a subset of the strains contained the gene, the percentage is given in parenthesis.

d A complete *efa1* gene (ca. 10 kb) was present as determined by PCR targeting the 3′, internal, and 5′ region, respectively [28].

e Only the 5′ region of *efa1* was present. Strains O145:H28/NM contained either complete *efa1* (two *stx*− and three *stx*+ strains) or only the 5′ *efa1* region (three *stx*− and seven *stx*+ strains).

f The *ter*-positive strains contained all genes of the *ter* gene cluster present in EHEC O157:H7 [25] (*terZ*, *terA*, *terB*, *terC*, *terD*, *terE*, and *terF*).

g Both genes were always present or absent together.

h The indicated *fliC* genotype was present in all strains of each respective serotype including strains that expressed the H antigen and nonmotile strains; n.p., not performed (because all strains expressed the H19 antigen).

### Comparison of phenotypes of *stx*−/*eae*+ *E. coli* and EHEC

The *stx*−/*eae*+ *E. coli* isolates shared with EHEC of the corresponding serotypes several diagnostically useful phenotypes (Table 3), but, in contrast to EHEC, their culture supernatants were not toxic to Vero cells (Table 3), the cell line that is sensitive to all Stx variants described until now. This suggests that these strains did not produce Stx encoded by *stx* gene(s) that might have been undetectable with our PCR protocol.

**Table 3:** Comparison of phenotypes of *stx*-negative and *stx*-positive *E. coli* strains of serotypes O26:H11/NM, O103:H2/NM, O121:H19, O145:H28/NM, and O157:H7/NM.

Phenotype^a^	Occurrence of the phenotype among *stx*-negative and *stx*-positive *E. coli* strains of serotype^b^											
	O26:H11/NM		O103:H2/NM		O121:H19		O145:H28/NM		O157:H7/NM		O157:NM	
	*stx*−	*stx*+	*stx*−	*stx*+	*stx*−	*stx*+	*stx*−	*stx*+	*stx*−	*stx*+	*stx*−	*stx*+
	(n = 13)	(n = 15)	(n = 4)	(n = 8)	(n = 1)	(n = 2)	(n = 5)	(n = 10)	(n = 2)	(n = 10)	(n = 15)	(n = 20)
Sorbitol fermentation	+	+	+	+	+	+	+	+	−	−	+	+
Rhamnose fermentation	−	−	+	+	+	+	+	+	+ (50)	+ (60)	−	−
Tellurite resistance	+ (92)	+ (93)	+ (25)	+ (13)	+	+	+	+	+	+	−	−
EHEC hemolysin	+	+	+	+	+	+	+	+	+	+	−	−
Cytolethal distending toxin V (CDT-V)	−	−	−	−	−	−	−	−	−	−	+ (87)^c^	+ (85)^c^
Shiga toxin	−	+	−	+	−	+	−	+	−	+	−	+

a The phenotypes were determined as described in Materials and Methods.

b NM, nonmotile; *stx*−, *stx*-negative; *stx*+, *stx*-positive. n, number of strains tested; +, all strains tested (n) expressed the phenotype; −, none of the strains tested (n) expressed the phenotype; if a subset of the strains expressed the phenotype, the percentage is given in parenthesis.

c The CDT-V titers were 1∶4–1∶16 in both *stx*-negative and *stx*-positive strains as determined by Chinese hamster ovary cell assay [26].

### Multilocus sequence typing (MLST) analysis of *stx*−/*eae*+ *E. coli* and EHEC

**Figure 1:**
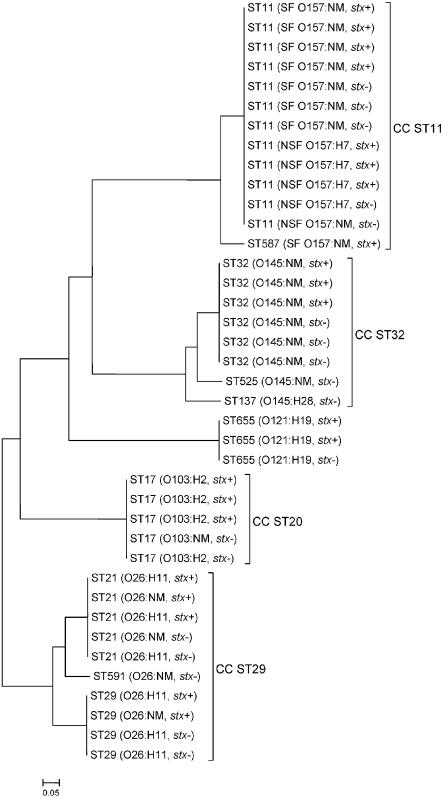
Phylogenetic relatedness of *stx*-negative and *stx*-positive *E. coli* strains within serotypes O26:H11/NM, O103:H2/NM, O121:H19, O145:H28/NM, and O157:H7/NM. Unrooted neighbor-joining tree was generated from allelic profiles of seven housekeeping genes (*adk*, *fumC*, *gyrB*, *icd*, *mdh*, *purA*, *recA*) [30] using the Phylip software package (http://evolution.genetics.washington.edu/phylip.html). ST, sequence type; CC, clonal complex (at least six identical alleles); NM, non-motile; *stx*, Shiga toxin-encoding gene; *stx*−, *stx*-negative; *stx*+, *stx*-positive; SF, sorbitol-fermenting; NSF, non-sorbitol-fermenting. Strains of serotype O121:H19 differ by at least 4 alleles from all known sequence types and have therefore no assigned clonal complex. Scale bar, 5% estimated evolutionary distance.

The phylogenetic relationships between the *stx*−/*eae*+ *E. coli* O26:H11/NM, O103:H2/NM, O121:H19, O145:H28/NM, and O157:H7/NM and EHEC of the same serotypes were determined by MLST analysis of randomly selected strains (Figure 1). The *stx*-negative and *stx*-positive strains of each serotype shared the same sequence type or at least six of the seven alleles investigated [30] and clustered therefore into the same clonal complex (Figure 1). In contrast, the *stx*-negative strains of the five different serogroups showed no close relationship based on their allelic profiles.

### Serological investigations

Serum samples were collected during the acute phase of HUS from 12 of 17 patients who shed *stx*−/*eae*+ *E. coli* O157 and from 10 of 26 patients who shed *stx*−/*eae*+ non-O157 strains. All 12 patients with *E. coli* O157 strains developed anti-O157 lipopolysaccharide (LPS) immunoglobulin M (IgM) antibodies. Among the 10 patients with non-O157 *E. coli* from whom serum samples were available, only the patient who shed *E. coli* ONT:H7 developed anti-O157 LPS IgM, suggesting a recent infection with *E. coli* O157 which probably precipitated the HUS. IgM antibodies to O157 LPS were not detected in the other nine sera from patients who shed *stx*−/*eae*+ *E. coli* O26:H11/NM (n = 5), O103:H2 (n = 1), O145:H28/NM (n = 2), and ONT:H6 (n = 1). Each of the five patients who excreted *stx*−/*eae*+ *E. coli* O26 had IgM antibodies to O26 LPS; presence of anti-O103 and anti-O145 LPS antibodies in the one and two patients, respectively, who shed *stx*−/*eae*+ *E. coli* strains of these serogroups could not be determined because of insufficient amount of the serum samples.

### Characterization of HUS patients who excreted *stx*−/*eae*+ *E. coli* but not other pathogens

Twenty of 43 HUS patients who shed *stx*−/*eae*+ *E. coli* as the only pathogens were males and 23 were females. Thirty-seven patients for whom information about age was available were children between 5 months and 9 years (mean age, 31.3 months; median age, 24 months). The mean age of these patients was significantly lower than that of patients who shed EHEC strains (range, 4 months to 64 years, mean, 34.6 months; median, 27 months) (*P* = 0.003; Mann-Whitney *U* test). None of the 43 patients who excreted *stx*−/*eae*+ *E. coli* strains and nine (2.0%) of the 440 patients who excreted EHEC died during the acute phase of HUS.

## Discussion

Stxs produced by EHEC are considered the major precipitants of the microvascular endothelial injury that underlies HUS [1], [12]. These toxins and their encoding genes are also the major targets exploited in the laboratory diagnosis of EHEC infections [2], [31]. Our finding of *stx*-negative *E. coli* strains that are closely related to EHEC as the only putative bacterial pathogens in stools of ∼5% of patients with HUS during a long-term study sheds therefore new light into microbiological, diagnostic, epidemiological, and clinical aspects of this disorder.

Several lines of evidence support the hypothesis that these *stx*-negative strains represent derivatives of original infecting EHEC that lost the ability to express Stx (EHEC-LST), in these cases because of *stx*-bacteriophage excision during infection. First, the spectrum of serotypes of the *stx*-negative isolates is similar to that of EHEC strains isolated from HUS patients (Table 1); in both cases, serotype O157:H7/NM (including both non-sorbitol-fermenting [NSF] and sorbitol-fermenting [SF] strains) is the most frequent, being followed by serotypes O26:H11/NM, O145:H28/NM, and O103:H2/NM (Table 1). These serotypes are generally not excreted by healthy subjects [32; H. Karch, unpublished data]. The absence of serogroup O111, which was found in 2.5% of EHEC-excreting patients, among the HUS-associated EHEC-LST isolates (Table 1) is probably because *stx*_1_, which is the most prevalent *stx* in EHEC O111 [33] is encoded within a defective prophage, which has been immobilized in the EHEC genome [34], preventing the *stx* loss by phage excision. Also, the ratio between SF and NSF EHEC-LST O157 isolates (88% vs. 12%) (Table 1), while in contrast to that observed between SF and NSF EHEC O157 isolated from HUS (26% vs. 74%) (Table 1), is proportional to the greater frequency with which the *stx* loss occurs in SF EHEC O157:NM [21], [22]. Second, the EHEC-LST isolates share with EHEC of the corresponding serotypes non-*stx* virulence and fitness genes and belong to the same MLST clonal complexes. This demonstrates a common phylogeny and conservation of variable genome regions in the two groups of organisms. Third, the possibility of *stx* loss from EHEC O26:H11/NM and SF EHEC O157:NM during the course of HUS has been proposed in our previous study based on closely related molecular characteristics of *stx*-positive and *stx*-negative isolates from the initial and follow-up stools, respectively [21]; the *stx* loss has been confirmed in vitro [16]. Fourth, the genomic loci where *stx*-converting bacteriophages integrate into the genomes of EHEC O26:H11/NM [16] and O157:H7/NM [14], [15] were unoccupied in all *stx*−/*eae*+ isolates of these serotypes indicating that the *stx* loss resulted from the excision of *stx*-converting phages. Altogether, these data strongly suggest that the *stx*−/*eae*+ *E. coli* strains isolated from HUS patients were EHEC-LST.

Although the design of the study does not allow to determine whether the *stx*−/*eae*+ *E. coli* strains could be primary pathogens that triggered the HUS in patients from whom they were isolated, this seems to be unlikely, taking into account the paradigm that HUS is caused by Stx [1], [2]. However, because they have unoccupied *stx*-bacteriophage integration sites, the *stx*−/*eae*+ *E. coli* O26:H11/NM and O157:H7/NM strains can be transduced with *stx*-harboring phages and converted thus to EHEC, at least in vitro [16]. Whether such an event can occur during infection and whether it could trigger HUS remains to be established. Moreover, conditions favoring lysogenic conversion or *stx* loss in vivo are poorly understood [18].

The ratio between EHEC-LST (5.5%) and EHEC (55.9%) isolated in our study is ∼1∶10. The finding that every 10^th^ patient with HUS, a condition that was most probably triggered by an EHEC infection, does not shed EHEC, but rather excretes EHEC-LST when stool is subjected to appropriate microbiological analysis, has important practical implications. First, the *stx* loss in an EHEC strain during infection can mislead epidemiological investigations because an *stx*-negative strain would not be, based on currently used criteria, considered to be epidemiologically related to *stx*-positive strains, even though of the same serotype. Therefore, the awareness of the possibility that a patient with HUS can excrete, in lieu of the original infecting EHEC, EHEC-LST which shares non-*stx* molecular characteristics with EHEC of the corresponding serotype, can assist epidemiologists to link correctly epidemiologically related cases, to identify the source of the infection and to trace modes of transmission. In such studies it is necessary to bear in mind that the loss of *stx*-harboring bacteriophages can alter pulsed-field gel electrophoresis patterns of the strains [15], [16], so that the epidemiologically related *stx*-positive and *stx*-negative strains can more or less differ in their fingerprints [16].

Second, microbiological identification of patients infected with EHEC O157:H7 early in illness is strongly associated with a good nephrologic outcome [1], probably because such an expeditious diagnosis prompts early volume expansion [35]. In patients excreting an EHEC-LST, the microbiological diagnosis may be delayed or the EHEC etiology of the disease is missed using tests that rely solely on the detection of *stx* genes or Stx production [31]. However, this concern might or might not be appropriate, depending on when in the course of the disease EHEC-LST replace the EHEC that almost certainly preceded the EHEC-LST. Whereas underdiagnosing EHEC-LST might not be clinically critical in patients with overt HUS who had already developed microvascular injury, the information about the presence of EHEC-LST in the stool (which may indicate continuing presence of the original EHEC in an amount undetectable by PCR *stx* screening) is of an important diagnostic value in patients with diarrhea, especially those with bloody diarrhea which often precedes HUS [1], [2], [5]. In such patients, a prompt diagnosis of EHEC-LST infection should alert the treating physician that the patient could develop HUS and should be monitored assiduously, receive isotonic volume expansion [35], and not be given antibiotics [36], [37] or antimotility agents [1], [38]. Clearly, we need better, and broader, microbiologic procedures to detect, in addition to *stx* or Stx, also non-*stx*/Stx EHEC targets.

How should EHEC-LST be detected? *eae*, which is present in the majority of EHEC isolated from HUS patients in Europe [2], [19] and the United States [7], [8], appears to be a quite appropriate additional diagnostic target. Specifically, based on our finding that *eae*-negative EHEC account for <4% of HUS-associated EHEC isolates in Germany [19] and assuming, based on the data from the present study, that *stx* loss occurs in ∼10% of the infecting EHEC, the using *eae* as a target to identify EHEC-LST would miss only ∼0.4% of such strains. This proportion of missed pathogens might be higher in regions where *eae*-negative EHEC account for a higher proportion of HUS isolates [10], [39]. In this case, the gene encoding the Stx-producing *E. coli* autoagglutinating adhesin (*saa*) [39], which is present in the majority of *eae*-negative EHEC associated with HUS [39], might be a suitable alternative to search for EHEC-LST. Optimal detection algorithms, and non-*stx* loci, depend on geographic and temporal-specific epidemiological trends, and for this reason it is necessary to continue microbiological surveillance. In this regard, it is critical to not abandon culture in favor of non-culture methodology, but to apply both modalities simultaneously to all stool specimens. In consideration of current epidemiology and etiology [1], [2], we believe that optimal diagnosis for both EHEC and EHEC-LST should consist of plating on sorbitol MacConkey (SMAC) agar (detection of NSF *E. coli* O157:H7/NM) and enterohemolysin agar (detection of the most frequent non-O157 serotypes based on the enterohemolytic phenotype) (Table 3) [9], [24], Stx or *stx* gene testing, and targeting *eae* (or *saa*) and *sfpA* (the latter for a specific detection of SF *E. coli* O157:NM) (Table 2) [23]. In culture-negative patients for whom serum samples are available, detection of antibodies against LPSs of the most frequent *E. coli* serogroups associated with HUS (both O157 and non-O157) can be an alternative approach to detect infection with both EHEC and EHEC-LST.

The application of *stx*/Stx-independent diagnostic strategies to identify EHEC-LST in HUS patients appears to be appropriate to consider for several reasons. First, stool samples from such patients are frequently collected only after HUS develops, i.e. ≥1 week after the onset of the prodromal diarrhea [1], as was true also for the majority of patients in our study. At this point in illness, EHEC might have been cleared [40] or *stx* might have been lost from infecting organisms [21]. In the latter case, using a non-*stx* diagnostic target such as *eae* or *saa* can still identify, with a high probability, the causative agent. Moreover, such diagnostic approaches would permit a prospective systematic clinical study to determine if *stx* loss by the infecting EHEC during the course of HUS might result in a less severe acute disease and/or a decreased rate of late sequelae.

Our study has two major limitations. First, although we systematically sought *stx*−/*eae*+ *E. coli* strains in stools that were negative for EHEC, as indicated by negative result of PCR screening for *stx* genes, we did not seek such strains in stool samples that contained EHEC. Stools that were *stx*-positive in PCR screening were only analyzed for *stx*-positive colonies. This approach rendered it impossible to determine if some of the patients had both *stx*−/*eae*+ *E. coli* strains and EHEC in the same stool, a situation that would indicate progressive loss of *stx* by the infecting EHEC population. Although poly-isolate analyses in other studies where a panel of markers were used including *stx* nucleic acid hybridization did not detect such a mixed population [41], [42], in these studies, stools were collected early in illness, and only five colonies were studied. Further studies targeting systematically both *stx* and *eae* (or other loci) in sequential stools from HUS patients are needed to determine the dynamic of *stx* loss during infection and to identify factors that could influence this process, such as serotype of the infecting EHEC, patient-related and/or environmental factors. Second, we do not have sufficient data to determine if antibiotics played a role in *stx* loss, as suggested in several experimental studies [14], [17].

In conclusion, at the time of microbiological analysis, an appreciable subset of patients with HUS shed no longer EHEC sensu stricto, but do excrete EHEC that lost *stx*. Diagnostic strategies need to be formulated to detect such pathogens and treating physicians should be immediately informed. Patients who shed such strains should be considered as potentially infected by EHEC and managed accordingly, at least until more data about the clinical significance of the EHEC-LST emerge. While not changing the paradigm that Stxs are the critical virulence determinant of EHEC responsible for HUS, our data do demonstrate the necessity of taking into account possible genetic changes of the pathogens during infection when developing appropriate diagnostic strategies and interpreting results of microbiological analyses.

## Materials and Methods

### Patients

During routine diagnostic work between 1996 and 2006, we sought *stx*−/*eae*+ *E. coli* and EHEC strains in stools (one stool per patient) from 787 epidemiologically unrelated patients with HUS. The patients were hospitalized in 23 pediatric nephrology centers in Germany and Austria described previously [5], with an extended period for patient enrollment until December 2006. Stools from patients who excreted *stx*−/*eae*+ *E. coli* strains and EHEC strains were collected between 5 and 14 days (median 9 days) and between 5 and 13 days (median 8 days), respectively, after the onset of prodromal diarrhea. The difference in the time of stool collection was not significant (*P* = 0.24, Mann-Whitney *U* test). HUS was defined as microangiopathic hemolytic anemia (hematocrit <30%, with evidence of the destruction of erythrocytes on a peripheral-blood smear), thrombocytopenia (platelet count <150,000/mm^3^), and renal insufficiency (serum creatinine concentration greater than the upper limit of the normal range for age) [36].

### Identification of *stx*−/*eae*+ *E. coli* in stools

*stx*−/*eae*+ *E. coli* strains were sought in parallel with EHEC as described [21], [23]. Briefly, stool samples enriched in Hajna broth were specifically enriched for *E. coli* O157 using immunomagnetic separation with Dynabeads anti-*E. coli* O157 (Invitrogen, http://www.invitrogen.com) and magnetically separated organisms were cultured on SMAC agar and cefixime-tellurite (CT)-SMAC agar (Oxoid, http://www.oxoid.com). To identify non-O157 *E. coli* strains, non-separated broth cultures were inoculated onto SMAC and enterohemolysin agar (Sifin, http://www.sifin.de). The overnight growth was harvested into saline and screened by PCR for *stx*_1_, *stx*_2_, *eae*, *rfb*_O157_ and *sfpA* genes [21], [23]; the latter two PCRs specifically detect *E. coli* O157 [23]. *stx*-positive stool cultures were further processed to isolate EHEC strains [21], [23]. From cultures which were *stx*-negative but *eae*-positive the *eae*-positive strains were isolated using colony blot hybridization with digoxigenin-labeled *eae* probe [21]. Among the 43 *stx*−/*eae*+ strains described here, 27 were isolated in this study and 16 (12 O157:H7/NM and four non-O157) in our previous studies [21], [22].

### Phenotypic methods

Resulting isolates were biochemically confirmed as *E. coli* (API 20 E; bioMérieux, http://www.biomerieux.de) and serotyped [43]. Fermentations of sorbitol and rhamnose were detected on SMAC and rhamnose MacConkey agar (Sifin), respectively [24]. The enterohemolytic phenotype was sought on enterohemolysin agar and resistance to tellurite on CT-SMAC [25]. Stx activity in culture supernatants and stool filtrates was detected using the Vero cell assay [19]. Production of cytolethal distending toxin (CDT) was determined using Chinese hamster ovary cells [26].

### Genotypic characterization

*eae*, presently known *stx* alleles [2], and other toxin (EHEC-*hlyA*, *cdt-V*) and adhesin (*iha, lpfA*_O26_, *lpfA*_O157/OI 141_, *lpfA*_O157/OI 154_, *sfpA*) genes were detected using established PCR protocols [19], [23], [24], [26], [27], [33], [44]. Moreover, the isolates were PCR tested for putative virulence genes located within O island 122 of EHEC O157:H7 strain EDL933 (*efa1*, *sen*, *pagC*) [11], [28], the *ter* gene cluster encoding tellurite resistance [25], and *irp2* and *fyuA*, which are components of the iron uptake system encoded on the high pathogenicity island [29]. *eae* genes were subtyped [45] and genotypes of the flagellin-encoding *fliC* gene were determined [9], [33]. The intact or occupied status of chromosomal loci that serve as phage integration sites (*yehV*, *wrbA*, *yecE*) was investigated using PCR primers and conditions described previously [14]–[16].

### MLST

MLST was performed by analyzing internal fragments of seven housekeeping genes (*adk*, *fumC*, *gyrB*, *icd*, *mdh*, *purA*, *recA*) [16], [30]. The alleles and sequence types were assigned in accordance with the *E. coli* MLST website (http://web.mpiib-berlin.mpg.de/mlst/dbs/Ecoli). The genetic relationships between different sequence types were determined using eBURST [46] and a phylogenetic tree based on neighbor-joining analysis was constructed using the Phylip package (http://evolution.genetics.washington.edu/phylip.html).

### Detection of additional classic bacterial enteric pathogens

*Salmonella*, *Shigella*, and *Yersinia* spp., and *Campylobacter jejuni* were sought using standard procedures [47]–[49].

### Serological investigation

IgM antibodies against the O157 and O26 LPS antigens were sought in sera from acute phase of HUS using an immunoblot [21].

### Statistical analysis

The non-parametric Mann-Whitney *U* (Wilcoxon) two-sample test for independent sample groups and OpenStat2 Software (http://www.statpages.org/miller/openstat/) were used for statistical analysis. *P* values <0.05 were considered significant.

## References

[1] Tarr PI, Gordon CA, Chandler WL (2005). Shiga toxin-producing *Escherichia coli* and the haemolytic uraemic syndrome.. Lancet.

[2] Karch H, Tarr PI, Bielaszewska M (2005). Enterohaemorrhagic *Escherichia coli* in human medicine.. Int J Med Microbiol.

[3] Siegler RL (2003). Postdiarrheal Shiga toxin-mediated hemolytic uremic syndrome.. JAMA.

[4] Lynn RM, O'Brien SJ, Taylor CM, Adak GK, Chart H (2005). Childhood hemolytic uremic syndrome, United Kingdom and Ireland.. Emerg Infect Dis.

[5] Gerber A, Karch H, Allerberger F, Verweyen HM, Zimmerhackl LB (2002). Clinical course and the role of Shiga toxin-producing *Escherichia coli* infection in the hemolytic-uremic syndrome in pediatric patients, 1997–2000, in Germany and Austria: a prospective study.. J Infect Dis.

[6] Tozzi AE, Caprioli A, Minelli F, Gianviti A, De Petris L (2000). Shiga toxin-producing *Escherichia coli* infections associated with hemolytic uremic syndrome, Italy, 1988–2000.. Emerg Infect Dis.

[7] Banatvala N, Griffin PM, Greene KD, Barrett TJ, Bibb WF (2001). The United States national prospective hemolytic uremic syndrome study: microbiologic, serologic, clinical, and epidemiologic findings.. J Infect Dis.

[8] Brooks JT, Sowers EG, Wells JG, Greene KD, Griffin PM (2005). Non-O157 Shiga toxin-producing *Escherichia coli* infections in the United States, 1983–2002.. J Infect Dis.

[9] Sonntag A, Prager R, Bielaszewska M, Zhang W, Fruth A (2004). Phenotypic and genotypic analyses of enterohemorrhagic *Escherichia coli* O145 strains from patients in Germany.. J Clin Microbiol.

[10] Elliott EJ, Robins-Browne RM, O'Loughlin EV, Bennett-Wood V, Bourke J (2001). Nationwide study of haemolytic uraemic syndrome: clinical, microbiological, and epidemiological features.. Arch Dis Child.

[11] Karmali MA, Mascarenhas M, Shen S, Ziebell K, Johnson S (2003). Association of genomic O island 122 of *Escherichia coli* EDL 933 with verocytotoxin-producing *Escherichia coli* seropathotypes that are linked to epidemic and/or serious disease.. J Clin Microbiol.

[12] Sandvig K (2001). Shiga toxins.. Toxicon.

[13] Richardson SE, Karmali MA, Becker LE, Smith CR (1988). The histopathology of the hemolytic uremic syndrome associated with verocytotoxin-producing *Escherichia coli* infections.. Hum Pathol.

[14] Shaikh N, Tarr PI (2003). *Escherichia coli* O157:H7 Shiga toxin-encoding bacteriophages: integrations, excisions, truncations, and evolutionary implications.. J Bacteriol.

[15] Bielaszewska M, Prager R, Zhang W, Friedrich AW, Mellmann A (2006). Chromosomal dynamism in progeny of outbreak-related sorbitol-fermenting enterohemorrhagic *Escherichia coli* O157:NM.. Appl Environ Microbiol.

[16] Bielaszewska M, Prager R, Köck R, Mellmann A, Zhang W (2007). Shiga toxin gene loss and transfer in vitro and in vivo during enterohemorrhagic *Escherichia coli* O26 infection in humans.. Appl Environ Microbiol.

[17] Zhang X, McDaniel AD, Wolf LE, Keusch GT, Waldor MK (2000). Quinolone antibiotic induces Shiga toxin-encoding bacteriophages, toxin production and death in mice.. J Infect Dis.

[18] Wagner PL, Waldor MK (2002). Bacteriophage control of bacterial virulence.. Infect Immun.

[19] Bielaszewska M, Friedrich AW, Aldick T, Schurk-Bulgrin R, Karch H (2006). Shiga toxin activatable by intestinal mucus in *Escherichia coli* isolated from humans: predictor for a severe clinical outcome.. Clin Infect Dis.

[20] Donnenberg MS, Tzipori S, McKee ML, O'Brien AD, Alroy J (1993). The role of the *eae* gene of enterohemorrhagic *Escherichia coli* in intimate attachment in vitro and in a porcine model.. J Clin Invest.

[21] Mellmann A, Bielaszewska M, Zimmerhackl LB, Prager R, Harmsen D (2005). Enterohemorrhagic *Escherichia coli* in human infection: in vivo evolution of a bacterial pathogen.. Clin Infect Dis.

[22] Friedrich AW, Zhang W, Bielaszewska M, Mellmann A, Köck R (2007). Prevalence, virulence profiles, and clinical significance of Shiga toxin-negative variants of enterohemorrhagic *Escherichia coli* O157 infection in humans.. Clin Infect Dis.

[23] Friedrich AW, Nierhoff KV, Bielaszewska M, Mellmann A, Karch H (2004). Phylogeny, clinical associations, and diagnostic utility of the pilin subunit gene (*sfpA*) of sorbitol-fermenting, enterohemorrhagic *Escherichia coli* O157:H^−^.. J Clin Microbiol.

[24] Bielaszewska M, Zhang W, Tarr PI, Sonntag AK, Karch H (2005). Molecular profiling and phenotype analysis of *Escherichia coli* O26:H11 and O26:NM: secular and geographic consistency of enterohemorrhagic and enteropathogenic isolates.. J Clin Microbiol.

[25] Bielaszewska M, Tarr PI, Karch H, Zhang W, Mathys W (2005). Phenotypic and molecular analysis of tellurite resistance among enterohemorrhagic *Escherichia coli* O157:H7 and sorbitol-fermenting O157:NM clinical isolates.. J Clin Microbiol.

[26] Janka A, Bielaszewska M, Dobrindt U, Greune L, Schmidt MA (2003). Cytolethal distending toxin gene cluster in enterohemorrhagic *Escherichia coli* O157:H- and O157:H7: characterization and evolutionary considerations.. Infect Immun.

[27] Toma C, Martinez Espinosa E, Song T, Miliwebsky E, Chinen I (2004). Distribution of putative adhesins in different seropathotypes of Shiga toxin-producing *Escherichia coli*.. J Clin Microbiol.

[28] Janka A, Bielaszewska M, Dobrindt U, Karch H (2002). Identification and distribution of the enterohemorrhagic *Escherichia coli* factor for adherence (*efa1*) gene in sorbitol-fermenting *Escherichia coli* O157:H^−^.. Int J Med Microbiol.

[29] Karch H, Schubert S, Zhang D, Zhang W, Schmidt H (1999). A genomic island, termed “high pathogenicity island”, is present in certain non-O157 Shiga toxin-producing *Escherichia coli* clonal lineages.. Infect Immun.

[30] Wirth T, Falush D, Lan R, Colles F, Mensa P (2006). Sex and virulence in *Escherichia coli*: an evolutionary perspective.. Mol Microbiol.

[31] Park CH, Kim HJ, Hixon DL (2002). Importance of testing stool specimens for Shiga toxins.. J Clin Microbiol.

[32] Beutin L, Marches O, Bettelheim KA, Gleier K, Zimmermann S (2003). HEp-2 cell adherence, actin aggregation, and intimin types of attaching and effacing *Escherichia coli* strains isolated from healthy infants in Germany and Australia.. Infect Immun.

[33] Zhang W, Mellmann A, Sonntag AK, Wieler L, Bielaszewska M (2007). Structural and functional differences between disease-associated genes of enterohaemorrhagic *Escherichia coli* O111.. Int J Med Microbiol.

[34] Creuzburg K, Kohler B, Hempel H, Schreier P, Jacobs E (2005). Genetic structure and chromosomal integration site of the cryptic prophage CP-1639 encoding Shiga toxin.. Microbiology.

[35] Ake JA, Jelacic S, Ciol MA, Watkins SL, Murray KF (2005). Relative nephroprotection during *Escherichia coli* O157:H7 infections: Association with intravenous volume expansion.. Pediatrics.

[36] Wong CS, Jelacic S, Habeeb RL, Watkins SL, Tarr PI (2000). The risk of the hemolytic-uremic syndrome after antibiotic treatment of *Escherichia coli* O157:H7 infections.. New Engl J Med.

[37] Zimmerhackl LB (2000). *E. coli*, antibiotics, and the hemolytic-uremic syndrome.. N Engl J Med.

[38] Cimolai N, Basalyga S, Mah DG, Morrison BJ, Carter JE (1994). A continuing assessment of risk factors for the development of *Escherichia coli* O157:H7-associated hemolytic uremic syndrome.. Clin Nephrol.

[39] Paton AW, Srimanote P, Woodrow MC, Paton JC (2001). Characterization of Saa, a novel autoagglutinating adhesin produced by locus of enterocyte effacement-negative Shiga toxigenic *Escherichia coli* strains that are virulent for humans.. Infect Immun.

[40] Tarr PI, Neill MA, Clausen CR, Watkins SL, Christie DL (1990). *Escherichia coli* O157:H7 and the hemolytic uremic syndrome: importance of early cultures in establishing the etiology.. J Infect Dis.

[41] Bokete TN, Whittam TS, Wilson RA, Clausen CR, O'Callahan CM (1997). Genetic and phenotypic analysis of *Escherichia coli* with enteropathogenic characteristics isolated from Seattle children.. J Infect Dis.

[42] Klein EJ, Stapp JR, Clausen CR, Boster DR, Wells JG (2002). Shiga toxin-producing *Escherichia coli* in children with diarrhea: a prospective point-of-care study.. J Pediatr.

[43] Prager R, Strutz U, Fruth A, Tschäpe H (2003). Subtyping of pathogenic *Escherichia coli* strains using flagellar (H)-antigens: serotyping versus *fliC* polymorphisms.. Int J Med Microbiol.

[44] Jelacic JK, Damrow T, Chen GS, Jelacic S, Bielaszewska M (2003). Shiga toxin-producing *Escherichia coli* in Montana: bacterial genotypes and clinical profiles.. J Infect Dis.

[45] Zhang WL, Kohler B, Oswald E, Beutin L, Karch H (2002). Genetic diversity of intimin genes of attaching and effacing *Escherichia coli* strains.. J Clin Microbiol.

[46] Feil EJ, Li BC, Aanensen DM, Hanage WP, Spratt BG (2004). eBURST: inferring patterns of evolutionary descent among clusters of related bacterial genotypes from multilocus sequence typing data.. J Bacteriol.

[47] Bopp CA, Brenner FW, Fields PJ, Murray PR, Baron EJ, Jorgensen JH, Pfaller MA, Yolken RH (2003). Escherichia, Shigella, and Salmonella.. Manual of clinical microbiology. 8th ed.

[48] Bockemühl J, Wong JD, Murray PR, Baron EJ, Jorgensen JH, Pfaller MA, Yolken RH (2003). Yersinia.. Manual of clinical microbiology. 8th ed.

[49] Nachamkin I, Murray PR, Baron EJ, Jorgensen JH, Pfaller MA, Yolken RH (2003). Campylobacter and Arcobacter.. Manual of clinical microbiology. 8th ed.

